# Load Quantification and Testing Using Flywheel Devices in Sports

**DOI:** 10.3389/fphys.2021.739399

**Published:** 2021-10-22

**Authors:** Sergio Maroto-Izquierdo, Javier Raya-González, José L. Hernández-Davó, Marco Beato

**Affiliations:** ^1^Department of Health Sciences, European University Miguel de Cervantes, Valladolid, Spain; ^2^Faculty of Health Sciences, Universidad Isabel I, Burgos, Spain; ^3^School of Health and Sports Sciences, University of Suffolk, Ipswich, United Kingdom; ^4^Institute of Health and Wellbeing, University of Suffolk, Ipswich, United Kingdom

**Keywords:** isoinertial, eccentric, performance, strength, team sports, football (soccer)

## Introduction

In the 90s, scientists were interested in finding a suitable technology for the mitigation of the negative effects associated with the absence of gravity during space flights (Kozlovskaya et al., 1990). Specifically, space flights have been associated with a decrement of musculoskeletal parameters in the order of 2% a week during the initial phase of a mission, which could represent a health and performance risk factor for astronauts (Thornton and Rummel, 1974; Kozlovskaya et al., 1990). Despite the reported benefits of resistance training in terms of muscle strength and mass gains, isotonic exercises require the utilization of heavy and frequently large devices (e.g., weight-stack machines or free weights), as well as these exercises are not gravity independent (Berg and Tesch, 1994). For such a reason, the first mechanical gravity independent device was developed to overcome these limitations, allowing crews to train in microgravity situations and limiting the deleterious effects of space flights (Berg and Tesch, 1994). After the first pioneering studies, sport scientists and later on, practitioners, understood the advantages offered by flywheel technology and they started to use it for training purposes such as performance development, injury prevention, and clinical rehabilitation (Askling et al., 2003; Tous-Fajardo et al., 2006; Tesch et al., 2017; Allen et al., 2021; Mcerlain-Naylor and Beato, 2021).

Flywheel exercise has some unique characteristics and offers several advantages compared to isotonic or traditional resistance exercises (Tesch et al., 2017; Madruga-Parera et al., 2020; Beato et al., 2021; Raya-González et al., 2021). Initially, sports scientists focused their attention on the benefits offered by the eccentric component of the flywheel exercise, which has been largely investigated (Douglas et al., 2017b; Suchomel et al., 2019a,b). Previous research suggests (although this theory is not completely accepted) that the eccentric contraction is capable to induce specific molecular adaptations for fast-twitch muscle fibers and protein synthesis (Lieber and Fridén, 1999; McHugh et al., 1999; Hody et al., 2019; Suchomel et al., 2019a), while from a mechanical perspective, it allows for higher force production and lower energy cost compared to concentric contraction (Zamparo et al., 2015; Douglas et al., 2017b). In addition, eccentric training can induce chronic neural (e.g., increased motor unit synchronization) and morphological (e.g., structure of the musculotendinous unit) adaptations (Douglas et al., 2017a; Hody et al., 2019; Beato and Dello Iacono, 2020; Beato et al., 2021). However, flywheel training benefits are not only related to the eccentric movement phase, but they are due to the combination of both concentric and eccentric contractions during the same exercise (Nuñez and Sáez de Villarreal, 2017; Beato and Dello Iacono, 2020). Although the concentric contraction in flywheel exercises have received lower interest compared to the eccentric contraction, it plays a key role in the obtainment of specific adaptations (both neural and morphological), and it is a necessary component for the development of the subsequent eccentric contraction (Norrbrand et al., 2008; Maroto-Izquierdo et al., 2017; Beato et al., 2019; Beato and Dello Iacono, 2020). For that reason, it is important to quantify kinetic and kinematic variables, such as velocity, power, or force, during both concentric and eccentric actions of the flywheel exercise to individually monitor and adjust training volume and intensity to fit the needs of each participant (Suchomel et al., 2019a; McErlain-Naylor and Beato, 2020; Piqueras-Sanchiz et al., 2020). Although the evidence to effectively prescribe flywheel training volume and intensity to achieve specific goals is still limited (Beato and Dello Iacono, 2020), load quantification provides an objective method to determine the precise number of repetitions per set or per session and the right inertia load needed to achieve the aims of training. For instance, if the aim of the session is to develop power, a given exercise can be stopped when concentric (or eccentric) peak power decrease due to fatigue, which can allow maintenance of similar power outputs in each set of the exercise. Therefore, the aim of this article is to provide the methodological bases for load quantification and testing using flywheel devices in sports. This paper is structured into three sections: (1) load quantification during flywheel exercise; (2) the use of flywheel devices for testing; and (3) limitations and future directions of load monitoring and testing using flywheel devices.

## Load Quantification During Flywheel Training

Load management is a key component for both athletes' programming and training monitoring process (Scott et al., 2016). Specifically, the intensity of resistance training programs has been traditionally prescribed relative to the maximum strength (based on the percentage of one-repetition maximum [1-RM]) (Suchomel et al., 2016). Another common method consists in the monitoring of concentric linear velocity of each repetition and, therefore, to provide a load-velocity profile for resistance exercises, which has enabled an optimized resistance training prescription with gravity-dependent exercises (Banyard et al., 2019). However, in the case of flywheel technology, it is not possible to determine the 1-RM since there is not a maximum load that can be lifted. Instead, during a flywheel exercise, the user needs to accelerate the inertia (discs) of the device during the concentric phase, which them will return the stored energy during the following eccentric phase of the exercise (Bollinger et al., 2018). During this braking phase, the user needs to progressively decelerate the flywheel (specific braking techniques can be used in this phase to accentuate the effort) and invert the movement returning to perform a concentric movement (Beato et al., 2020a). Previous research has reported that a near-maximal concentric contraction is a necessary condition to obtain a demanding eccentric contraction (Beato and Dello Iacono, 2020). Therefore, considering the maximum nature of each concentric contraction together with the need to know the magnitude of the load during the eccentric contraction (i.e., eccentric:concentric ratio [E:C ratio]), practitioners should consider to quantify both outputs during any given exercise (Carroll et al., 2019; McErlain-Naylor and Beato, 2020). The flywheel exercise intensity depends on the inertial discs used (i.e., modifying the moment of inertia) and mechanical characteristics of the device (e.g., cylinder type). In this sense, it is well-known that flywheel exercises using lower inertial loads generate high velocity movements (shorter eccentric-concentric coupling time), which were suggested to favor quick and explosive muscle-related adaptations, whereas exercises using higher inertial loads generate lower velocities and were suggested to favor greater force production and maximal strength adaptations (Martinez-Aranda and Fernandez-Gonzalo, 2017; Carroll et al., 2019). Moreover, recent research has shown that both exercise intensity and E:C ratio also depend on the axis type (i.e., horizontal regular cylinder or vertical conical cylinder) and radius of the flywheel device used (Núñez et al., 2020), showing higher mechanical outputs and larger eccentric-overload when horizontal cylinders were used compared to their vertical conical counterparts (Muñoz-López et al.,2021). In addition to inertial discs selected and the characteristics of the flywheel device used, the movement velocity, which is primary depended of the participants' strength and physical characteristics, are key determinant of both concentric and eccentric outputs (e.g., power) of a flywheel exercise (Maroto-Izquierdo et al., 2019; McErlain-Naylor and Beato, 2020).

Recently, the use of inertia–power and inertia–velocity relationships have been investigated (Carroll et al., 2019; McErlain-Naylor and Beato, 2020). It has been reported that when performing any given flywheel exercise both concentric and eccentric velocity decrease while inertial load increases (McErlain-Naylor and Beato, 2020). But this is not the case for mechanical power, which highlights that, although peak power is often used as the main parameter to quantify flywheel exercise intensity (e.g., during a squat), power output should not be used as unique parameter to evaluate exercise intensity (McErlain-Naylor and Beato, 2020; Worcester et al., 2020). Recent evidence suggests that the monitoring of velocity (mean and peak) should be preferred to power, but although concentric and eccentric velocity can be used to effectively prescribe exercise intensity (Carroll et al., 2019; McErlain-Naylor and Beato, 2020), their accurate monitoring is quite complicated and requires the use of advanced technologies (e.g., 3D motion capture) that are not commonly available to practitioners. In most cases, practitioners use integrated rotary encoders, which provide information about the angular velocity of the flywheel (Bollinger et al., 2018; Weakley et al., 2019), but it should be considered the existence of a dissonance between what happens on the axis (i.e., angular velocity in a conical cylinder flywheel device) (Núñez et al., 2020; Sabido et al., 2020), and what happens at kinematic level while the participant is performing the exercise, which limited the use of velocity for such purposes. Although angular velocity can be converted to linear velocity mathematically, this relationship relies on a known radius (which can vary within some devices) and the lack of slack in cable/rope. Therefore, further studies are warranted to determine other feasible load quantification strategies (e.g., linear velocity quantification).

In daily practice (e.g., strength training in clinical and sport contexts) the two most common parameters used to modulate and monitor the load intensity are: the moment of inertia (i.e., inertia load—the number and combination of discs) and the power outputs (i.e., concentric, eccentric, and E:C ratio). For such a reason, practitioners generally determine the inertia–power profile of a given flywheel exercise, which allows the selection of the optimal inertia load for the subject (e.g., power output maximization) (Carroll et al., 2019; McErlain-Naylor and Beato, 2020; Piqueras-Sanchiz et al., 2020; Worcester et al., 2020); and it can be used as a feasible and reliable method for assessing training adaptations and progress (Núñez et al., 2018).

## The Use of Flywheel Devices for Testing

Previous researchers have proposed some inertial incremental tests to determine the inertial load in which the highest concentric peak power was developed (de Hoyo et al., 2014; Maroto-Izquierdo et al., 2020). In most of them, participants are requested to perform between 4 and 6 sets of 4 maximal repetitions with different inertial loads (between ~0.01 and ~0.10 kg·m^2^) with a 3-min recovery between sets, until finding the inertial load in which concentric (or eccentric) peak power decreases compared to the previous one. This protocol was used for determining the inertia-power profile of a flywheel squat exercise, which reliability was recently reported for force and mean velocity results (intra-class correlation coefficient [ICC] > 0.9) (Spudić et al., 2020). However, this type of protocol presents some limitations, for instance, the power outputs recorded during this testing protocol may be affected by accumulated fatigue (during previous sets), therefore, strategies such as to prescribe an adequate recovery between sets and randomization of the order of the inertias used should be considered during the test. Additionally, familiarization with the exercise selected (e.g., squat) and a test-retest procedure to confirm data reliability should also be considered by practitioners. Moreover, power outputs do not drastically change with the variation of the inertial load because power derives from the combination of force and velocity (Carroll et al., 2019; McErlain-Naylor and Beato, 2020), which represents a further complication for practitioners. Furthermore, the peak power only represents a single moment in time and provides no kinetic or kinematic information about the rest of the movement (or even the position in which this peak occurred). The most common power outputs used by practitioners to evaluate chronic adaptations are concentric and eccentric power and E:C ratio, but while concentric and eccentric power outputs have demonstrated excellent reliability, this has not been the case for E:C ratio, which limited its application for long-term training assessments (Sabido et al., 2018; Beato et al., 2020b; Piqueras-Sanchiz et al., 2020). Recently, a flywheel squat test protocol has been developed to evaluate such adaptations in sports. It consists of 3 sets of 6 repetitions (two initial repetitions were added to attain the initial momentum) using an inertial load of ~0.06 kg^.^m^2^, where the average of the peak power outputs of the six repetitions of the second and third sets are recorded, while the first set is excluded for reliability reasons—see the following paper for further detail on the protocol, data analysis, and interpretation (Beato et al., 2020b). This test reported excellent test-retest reliability for concentric and eccentric power outputs (*ICC* = 0.94–0.95) and reported the existence of a significant linear relationship with isokinetic concentric and eccentric parameters (i.e., peak torque). Practitioners may consider using this validated test in daily practice, which may offer useful information for flywheel training periodization.

## Limitations and Future Directions of Load Monitoring and Testing Using Flywheel Devices

Flywheel load monitoring has been studied in the last years and some papers have reported the utility to evaluate inertia–power and inertia–velocity relationships (Carroll et al., 2019; McErlain-Naylor and Beato, 2020). However, both these approaches present some advantages and limitations, therefore future research is needed to better understand how to efficiently monitor the training load (e.g., power output, movement velocity, force output) in users, which can play a key role in flywheel training. A second limitation can be found in the existing flywheel testing procedures because only one study has currently reported both validity and reliability of a protocol (i.e., flywheel squat) (Beato et al., 2020b). Therefore, further research is needed to verify the existing relationship between flywheel test results and sport-specific actions such as jumping, sprinting, changing of direction, etc. Additionally, more information is needed around the length and modality of the familiarization process to obtain reliable data during both training load monitoring and testing. Based on previous research, it seems possible to become familiarized with flywheel exercises after 2–3 sessions (Sabido et al., 2018; Beato and Dello Iacono, 2020), but the real familiarization duration is participant dependent, therefore a much greater variability can be expected, which required an individualized process.

Lastly, the majority of available flywheel devices use rotary encoders incorporated into flywheel devices to determine concentric and eccentric power outputs, but users should take into account that some of these encoders may not be sufficiently accurate to assess performance variables and therefore their reliability should be evaluated before their use (Bollinger et al., 2018; Weakley et al., 2019). Future research should evaluate the use of 2D video technology or wearable technology (i.e., accelerometers) for the assessment of kinematic variables.

## Conclusions

This article provides a clear summary of the background and rationale of the use of flywheel exercise in sports, starting from the first pioneering studies to the latest evidence about load monitoring and testing using flywheel devices. This study reports that both inertia–power and inertia–velocity relationships can be used to design the profile of a given flywheel exercise, which can be used for assessing training adaptations and progress. Moreover, the optimal inertial load can be assessed using a progressive testing protocol evaluating the peak power, this approach allows to individualize the load for each participant based on their own physical characteristics. Lastly, a flywheel squat test protocol developed to evaluate adaptations has been recently validated—reporting excellent test-retest reliability for concentric and eccentric power outputs.

## Author Contributions

All authors listed have made a substantial, direct and intellectual contribution to the work, and approved it for publication.

## Conflict of Interest

MB declares to have received financial support for his research from a private company producing flywheel devices in 2020. The remaining authors declare that the research was conducted in the absence of any commercial or financial relationships that could be construed as a potential conflict of interest.

## Publisher's Note

All claims expressed in this article are solely those of the authors and do not necessarily represent those of their affiliated organizations, or those of the publisher, the editors and the reviewers. Any product that may be evaluated in this article, or claim that may be made by its manufacturer, is not guaranteed or endorsed by the publisher.
